# Construction of RNA m6A profiles in liver tissue of mice in sepsis-induced liver injury based on m6A MeRIP-seq and RNA-seq

**DOI:** 10.1186/s40001-025-02985-7

**Published:** 2025-08-08

**Authors:** Li Li, Penghui Li, Yuhua He, Ziyi Xu, Yitao Fan, Xueying Jia, Yingru Liu, Lijie Qin

**Affiliations:** 1 Department of Emergency, Henan Provincial People’s Hospital, People’s Hospital of Zhengzhou University, Zhengzhou, 450003 Henan China; 2 Henan Key Laboratory of Cancer Epigenetics, Cancer Institute, The First Affiliated Hospital, College of Clinical Medicine, Medical College of Henan University of Science and Technology, Luoyang, 471003 Henan China; 3 Department of Infectious Diseases, The First Affiliated Hospital of Zhengzhou University, Zhengzhou University, No. 1 Jianshe East Road, Erqi District, Zhengzhou, 450052 Henan China

**Keywords:** Sepsis, Liver injury, N6-methyladenosine, METTL3, MeRIP-seq

## Abstract

Sepsis-induced liver injury (SILI) indicates liver functional or structural impairment occurring during sepsis and is one of the common complications of sepsis. N6-methyladenosine (m6A) modification plays a significant role in the pathological processes of SILI, but its specific mechanisms remain unclear and require further elucidation. In this study, an experimental sepsis model exhibiting characteristic symptoms and hepatic damage was established through cecal ligation and puncture (CLP) surgery. Comprehensive analyses of hepatic tissues from CLP-treated and Sham operation mice through RNA Sequencing (RNA-Seq) and Methylated RNA Immunoprecipitation Sequencing (MeRIP-Seq) methodologies revealed distinct m6A peaks and differentially expressed genes (DEGs). A total of 2002 m6A peaks were detected (|log2FC|≥ 1, *p* < 0.05). Our analyses demonstrated that 71% of m6A peaks clustered within the coding sequences (CDS) and 3' untranslated region starts (3' UTR) of transcripts. When compared with the Sham group, the CLP group showed an increase of 1741 DEGs and a decrease of 1815 DEGs. And 458 genes exhibited both m6A modifications and changes in mRNA levels. Functional enrichment assessments through Gene Ontology (GO) and Kyoto Encyclopedia of Genes and Genomes (KEGG) analyses revealed that m6A-modified hepatic genes in septic mice were significantly enriched in the cytoplasm, nucleus and protein binding, which mainly involved in regulating cell survival, immune responses, inflammatory responses and other related pathways. The results of Cell Counting Kit-8 (CCK8) assay and 5-ethynyl-2'-deoxyuridine (EdU) assay showed that the proliferation ability of liver cell lines was enhanced after knocking down m6A regulatory factor METTL3, which indicates the m6A modifications play an important role in the proliferation ability of normal liver cells. This study validated the presence of m6A-epitranscriptomic modifications and their regulatory roles in sepsis-affected hepatic systems, establishing comprehensive m6A landscapes in septic liver tissues.

**Supplementary Information:**

The online version contains supplementary material available at 10.1186/s40001-025-02985-7.

## Background

Sepsis stands as a perilous complication frequently encountered in the wake of infections, traumatic injuries, and extensive surgical interventions, posing as a primary threat to life in intensive care units (ICUs) [1]. Its essence lies in severe, acute organ dysfunction triggered by the body’s improper response to infection [2]. According to the latest global statistics, sepsis has ascended to the ranks of a significant global health crisis, with alarming statistics indicating 48.9 million cases and claiming 11 million lives annually [3]. The intricate pathophysiology behind sepsis involves a cascade of inflammatory mediators, an overly aggressive immune system activation, and the subsequent manifestation of multiple organ dysfunction syndrome [4]. Notably, studies have highlighted a correlation between numerous single nucleotide polymorphisms in Toll-like receptors (TLRs) and their signaling components, shedding light on potential genetic factors influencing individual susceptibility to sepsis [5].

As an uncontrolled systemic inflammatory response, the liver coordinates immunological defenses through pathogen elimination, synthesizing acute-phase reactants and inflammatory mediators, while implementing metabolic reprogramming to address inflammatory challenges [6, 7]. Functioning as a critical component in antimicrobial defense, the liver demonstrates dual functionality by facilitating pathogen removal while simultaneously initiating immunomodulatory mechanisms that prevent excessive immune activation. Impairment of this delicate equilibrium results in sepsis-associated hepatic damage and elevated mortality rates in septic patients [8]. Research demonstrates that systemic inflammation profoundly impacts hepatic physiology, causing substantial modifications in transcriptional and translational profiles, activating pro-inflammatory signaling cascades, while suppressing essential homeostatic processes including metabolic regulation, xenobiotic transformation, and biliary transport mechanisms [9–11]. These pathophysiological changes substantially modify the composition of plasma proteins and metabolites during systemic inflammatory states [12]. Despite the recent advancements in the understanding of SILI, its underlying mechanisms, diagnosis, and treatments remain significant issues and its high morbidity and mortality continue to pose a substantial threat to global public health [4, 13].

The recent epigenetic research underscores the crucial role of diverse modifications in gene expression regulation, with N6-methyladenosine (m6A) emerging as a prominent RNA modification. This dynamic and reversible process governs mRNA stability, degradation, and post-transcriptional translation. Consequently, m6A modifications significantly impact gene expression, influencing vital biological functions such as energy metabolism, immune responses, inflammation, and programmed cell death [14, 15]. The regulation of m6A modifications is orchestrated by the following three specific proteins: the methyltransferase, termed the “writer” (comprising METTL3, METTL14, WTAP, etc.), the demethyltransferase, termed the “eraser” (including FTO, ALKBH5, etc.), and the m6A-binding protein, termed the “reader” (encompassing YTHDF1, YTHDF2, YTHDF3, etc.) [16]. The dynamic changes of these three regulatory proteins render m6A RNA modification pivotal in sepsis. Research has demonstrated that m6A modifications can affect septic progression and prognosis of the disease by modulating gene expression and immune responses [17]. After the advent of methylated RNA immunoprecipitation sequencing (MeRIP-seq), the interaction between the m6A transcriptome and diseases has been the subject of extensive investigation [18].

Recent studies have shed light on how sepsis triggers liver injury, utilizing scRNA sequencing analysis to uncover the underlying mechanisms. Macrophages are recruited during sepsis, and release various inflammatory factors such as TNF, IL-1β and IL-6, along with chemokines like CCL6 and CD14, and the transcription factor NF-κB1. This cascade initiates inflammatory responses in the liver. In addition, extensive lymphocyte apoptosis and abnormal neutrophil recruitment contribute to immune dysfunction [19]. Notably, METTL3, originally isolated from HeLa cells in 1997 by Boker et al. has been established as a crucial component of the N6-adenylate methyltransferase subunit [20]. The m6A modifications caused by METTL3 may promote the development of SILI by regulating the immune metabolic network of the liver axis and the phenotypic transformation of macrophages [21].

In this study, we compared the m6A transcriptome profiles of sepsis and normal liver tissue and investigated the distinct m6A modification patterns between the two groups. Results indicated the presence of a coexpression between the differentially expressed m6A-modified genes and DEGs. When m6A regulatory factor METTL3 was knocked down in AML-12 and THLE-2 cells, the proliferation ability of normal liver cells was enhanced. It was verified that METTL3 has a significant impact on the proliferation ability of normal liver cells, which indicates the m6A methylation modification makes a big difference to septic liver tissues. These discoveries help in further clarifying the function of m6A in the development of SILI.

## Methods

### Mouse model of sepsis-related liver injury

Male C57BL/6 J mice, aged between 7 and 9 weeks, were purchased from Beijing Vital River Laboratory Animal Technology Co., Ltd. (Beijing, China). Prior to initiating any experiments, the mice were acclimated for 1 week in an environment featuring a well-regulated 12-h light and 12-h dark cycle, along with controlled temperature and humidity conditions to minimize stress and ensure consistency across experimental groups. The mice were randomly divided into two groups, with five mice in each group. One group underwent cecal ligation and puncture (CLP) surgery, a commonly used model to induce polymicrobial peritonitis. This surgical procedure involved ligating the cecum 1 cm from its distal end using a 4–0 suture thread and then puncturing it with a 22-gauge needle at a point midway between the ligation and the cecum tip. The CLP surgery was adopted from an established protocol [22]. Mice in Sham group underwent a similar surgical procedure but without the ligation and puncture of the cecum. Following the surgical procedures, all mice were administered 1 ml of sterile lactated Ringer’s solution subcutaneously to prevent dehydration and aid in their recovery. All mice were given access to food and water ad libitum.

Twenty four hours post-surgery, we evaluated liver injury severity by measuring serum markers. In addition, we obtained three liver tissue samples from both the CLP and Sham groups for RNA Sequencing (RNA-Seq) and MeRIP-seq analyses. All the mouse experiments were approved by the Ethics Committees of the First Affiliated Hospital of Zhengzhou University (Approval No. 2025-KY-0544-001).

### Hematoxylin–Eosin (HE) staining

The extent of liver injury was assessed by HE Staining. Serum aspartate aminotransferase (AST) and alanine aminotransferase (ALT) levels were measured by the respective kits (Jiancheng, Nanjing, China) according to the manufacturer’s instructions. HE Staining is a commonly used technique to examine liver tissue damage in medical research. Mouse livers, as subjects in this study, were typically fixed in 4% paraformaldehyde to preserve their structures. Fresh liver tissues were then processed through a series of gradient ethanol solutions for dehydration, followed by embedding in paraffin for sectioning. The paraffin-embedded tissue blocks were meticulously cut into sections measuring approximately 4 μm in thickness subjected to a series of processing steps, including deparaffinization with xylene, rehydration through graded ethanol solutions, and staining with HE. The HE-stained sections independently examined by two pathologists who were blinded to this study to ensure the objectivity of the results.

### RNA-seq

Total RNA was isolated with Trizol reagent (Thermo Fisher, 15596018), followed by quality assessment of RNA concentration and purity through the Bioanalyzer 2100 system combined with RNA 6000 Nano LabChip Kit (Agilent, USA, 5067–1511). Only RNA specimens demonstrating integrity values (RIN) exceeding 7.0 were considered suitable for subsequent library preparation. mRNA isolation from 5 μg total RNA was accomplished through selective binding with Dynabeads Oligo (dT) (Thermo Fisher, USA). Fragmentation of mRNA was achieved through thermal disruption at 94 °C for 5–7 min employing divalent cations via the Magnesium RNA Fragmentation Module (NEB, e6150, USA). The prepared libraries underwent sequencing analysis on an Illumina NovaseqTM6000 platform (LC-Bio Technology CO., Ltd., Hangzhou, China) utilizing paired-end 150 bp sequencing configuration.

The initial processing of raw sequencing data involved trimming low-quality bases with fastp (version: fastp-0.19.4), followed by ribosomal RNA removal through RNAcentral to generate high-confidence clean reads. Genome alignment was performed through HISAT2 (version: HISAT2-2.2.1) for reference genome matching, with subsequent read mapping executed on the assembled genome. Transcript assembly and quantification were achieved using StringTie (version: Stringtie-2.1.2) under standard parameters, complemented by ballgown for comprehensive expression level estimation. Transcript abundance was quantified through FPKM (fragment per kilobase transcript per million reads) calculations to evaluate mRNA expression patterns. DEGs analysis was conducted with DESeq2 for group comparisons and edgeR for pairwise sample evaluations, applying stringent thresholds of false discovery rate (FDR) < 0.05 and minimum fold change (FC) ≥ 2 for identifying significant DEGs. Subsequent functional characterization of these genes involved comprehensive GO term analysis and KEGG pathway analysis to elucidate biological processes and molecular functions.

### MeRIP-seq

Total RNA was extracted using TRIzol reagent (Invitrogen, CA, USA), followed by determination of its concentration and purity through a NanoDrop ND-1000 spectrophotometer (NanoDrop, Wilmington, DE, USA). Further validation of RNA quality was carried out using the Bioanalyzer 2100 system (Agilent, CA, USA), ensuring that the RNA integrity number (RIN) was above 7.0. Polyadenylated RNA was purified from 30 micrograms of total RNA using Dynabeads Oligo (dT) 25-61005 (Thermo Fisher Scientific, USA). Subsequently, the RNA was fragmented using the NEBNext^®^ Magnesium RNA Fragmentation Module (Catalog No. E6150S, USA). For the enrichment of m6A, 90% of the fragmented RNA underwent immunoprecipitation with m6A-specific antibodies (Synaptic Systems, Catalog No. 202003, Germany) in an IP buffer consisting of 50 mM Tris–HCl, 750 mM NaCl, and 0.5% Igepal CA-630. This incubation took place overnight at 4°C. Ultimately, the sequencing analysis was conducted using the Illumina NovaSeq^™^ 6000 platform provided by LC-Bio Technology Co., Ltd., in Hangzhou, China. The reads were paired-end with a length of 150 basepairs, ensuring high-quality data for downstream analysis.

The initial processing of sequencing data involved trimming low-quality bases with fastp (version 0.19.4), followed by ribosomal RNA depletion through RNAcentral to generate refined reads. Alignment of Input and IP sample sequences to the Homo sapiens GRCh38 genome assembly was performed using HISAT2 (version 2.2.1). The exomePeak R package facilitated detection of methylation variations, with subsequent peak annotation conducted through ANNOVAR software (statistical significance threshold set at *p* < 0.05). HOMER software executed motif characterization of the detected peaks. For transcriptome analysis, edgeR package identified differentially expressed mRNAs through dual criteria: absolute log2 FC exceeding 1 and p-value below 0.05. Functional interpretation of genes linked to methylation variations was achieved through comprehensive GO term analysis and KEGG pathway analysis.

### Cell culture and transfection

Normal mouse hepatocyte cell lines AML-12 and normal adult liver epithelial THLE-2 cell lines were purchased from the Chinese Academy of Sciences (Shanghai). AML-12 cell lines were cultured in Roswell Park Memorial Institute 1640 (RPMI-1640 medium) (SC101-01, SEVENbio, China) supplemented with 10% fetal bovine serum (FBS) (FSD500, Excell bio, China) and 1% penicillin–streptomycin (SC118, SEVEN bio). THLE-2 cell lines were cultured in Dulbecco’s Modified Eagle’s Medium (DMEM) (SC102-02, SEVENbio, China) containing 10% fetal FBS (FSD500, Excell bio, China) and 1% penicillin–streptomycin (SC118, SEVEN bio, China). Both cell lines were continuously cultured at 37 °C in a sterile humidified incubator with 5% CO2. For cell transfection, puromycin-resistant lentiviral plasmid vector (pLKO.1-puro) and short RNA against METTL3 (shMETTL3-1, shMETTL3-2) and negative control (shNC) were obtained from Genomeditech (Shanghai). Stably transfected cell lines were selected by puromycin (2 μg/mL) (ST551, Beyotime, China).

### Quantitative real-time polymerase chain reaction (RT-qPCR)

Total RNA in hepatic cells was extracted using FlysisAmp Cells Lysis Kit (CL101-01, Vazyme, China) and reverse-transcribed to complementary DNA (cDNA) using FlysisAmp Cells-to-cDNA Kit (CL111-01, Vazyme, China). The reaction mixture of cDNAs and SYBR Green PCR Kit (Q711, Vazyme, China) was subjected to RT-qPCR to assess mRNA expression in the real-time PCR instrument (CFX Opus 384, Bio-Rad, USA). GAPDH was used as a housekeeping gene for standardization.

### 5-ethynyl-2'-deoxyuridine (EdU) assay

Cell proliferation was assessed by EdU assay according to the manufacturer’s protocol provided with the EdU cell proliferation Kit (C0075S, Beyotime). Briefly, 4000 AML-12 and THLE-2 cells were transferred into 96-well plates and subsequently incubated with EdU reagent for 2 h. For fixing and permeabilizing the cells, they were incubated with 4% formaldehyde solution (1 mL, G1101, Servicebio) and PBS containing 0.3% Triton X-100 (1 mL, GC204003, Servicebio) for 10 min at room temperature respectively. After washing the cells, a click reaction mixture was introduced to stain cells in the dark, then the Hoechst 33342 solution was added to stain nucleus, followed by the observation under the fluorescence microscope. The proliferation rate was calculated by the ratio of cells positive for EdU to those positive for Hoechst 33342 using ImageJ software.

### Cell counting kit-8 (CCK8) assay

Cell proliferation was assessed using the CCK8 assay from Sigma-Aldrich, following the manufacturer’s protocol. Cells were plated in 96-well plates at 1000 cells/well. CCK8 solution (10 μL) was added to each well and incubated for 2 h. Absorbance readings at 450 nm were used to plot cell proliferation curves.

### Statistical analysis

Statistical analysis was conducted using the R package edgeR for data from three or more independent experiments. Student’s *t* tests were performed to compare the Sham and CLP groups. Data are expressed as mean ± standard deviation, with statistical significance set at *p* < 0.05 for all comparisons.

## Results

### Sepsis induced liver injury

**Fig. 1:**
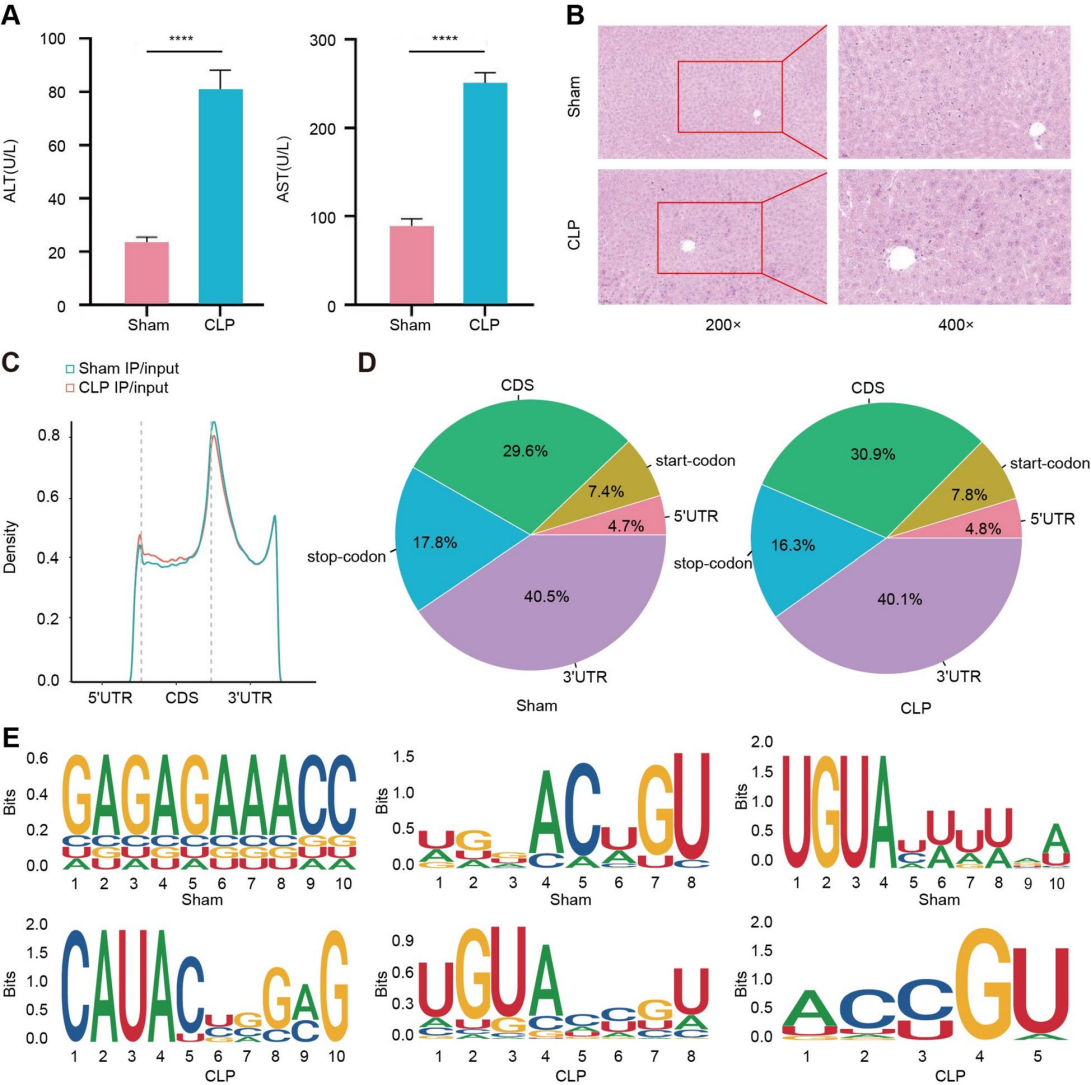
Establishment of sepsis mouse model and differential methylated m6A peaks between Sham and CLP groups based on MeRIP-seq data. **A** The concentrations of ALT and AST in the blood of Sham and CLP groups have increased at 24 h after treatment; **B** HE Staining results revealed liver tissues in the CLP group are characterized by disordered cell arrangement, interstitial edema, hemorrhage and inflammatory cell infiltration, while liver tissues in Sham group is normal; **C** Peak density chart showed that the levels of m6A peaks in the CLP group were significantly higher than that in the Sham group, while there were few differences in other functional elements of transcript variants; **D** Pie charts of m6A peaks in the 3′-UTR, CDS, and 5′-UTR regions of transcript variants in Sham and CLP groups showed that the m6A peaks in CLP group were distributed in the CDS and 3′ UTR mainly; **E** The top three Motif maps based structure conservation

The mice exhibited characteristic symptoms of sepsis, including lethargy, piloerection, and diarrhea after CLP treatment. Analysis of serum biomarkers revealed compromised hepatic function in the mice at 24 h post-CLP treatment, as evidenced by a significant elevation in ALT and AST levels (Fig. 1A). Correspondingly, histopathological examination of the damaged liver tissues revealed disordered cell arrangement, interstitial edema, hemorrhage and inflammatory cell infiltration in the CLP-treated mice when compared with the Sham group (Fig. 1B). Collectively, these findings indicate the successful establishment of a murine model for SILI. Subsequently, to reveal the expression profiles of RNA m6A methylation modification in liver tissues of mice with SILI, liver tissue samples were collected from both the Sham-operated group and the CLP group were processed for RNA-seq and MeRIP-seq analyses.

### MeRIP-seq was used to identify differentially methylated m6A peaks in sepsis

We obtained the m6A methylation modification characteristics of genes in the liver tissue of septic mice through library construction, MeRIP-seq sequencing and bioinformatics analyses of the samples. A total of 2002 m6A peaks were detected (|log2FC|≥ 1, *p* < 0.05). The m6A peaks appear at three different coordinates: the coding sequences (CDS), 5’ untranslated region (5'UTR), and 3'untranslated region starts (3'UTR). In the CDS region, the degree of methylation in the CLP group was significantly higher than that in the Sham group, while there were few differences in other regions (Fig. 1C). To systematically assess the degree of enrichment, the m6A peaks in both the Sham group and the CLP group were assigned to five functional elements: start codon, CDS, stop codon, 5'UTR, and 3'UTR. 71% of the m6A peaks in CLP group were located in the CDS and 3'UTR approximately, while 29% were located in the start codon, stop codon, and 5'UTR (Fig. 1D). Based on the structural conservatism, we have listed the top three motifs related to the m6A peaks among the differences (Fig. 1E).

### RNA-seq was used to identify DEGs in sepsis

**Fig. 2:**
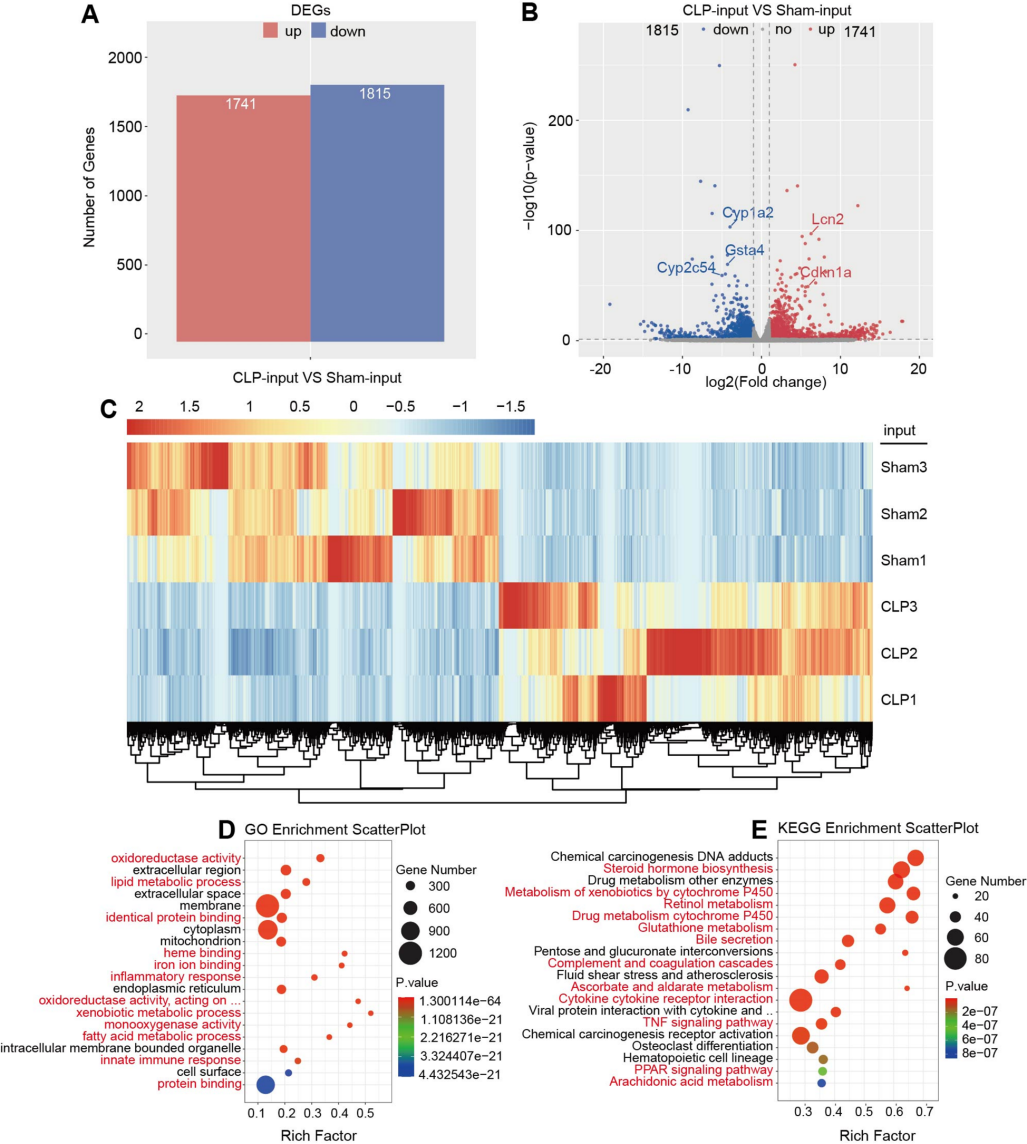
Differential gene expression between Sham and CLP groups based on RNA-seq data. **A** Bar chart of upregulated and downregulated genes; **B** Volcano plot of differential gene expression; **C** Heatmap of DEGs; **D** Bubble chart of GO enrichment of the top 20 DEGs; **E** Bubble chart of KEGG enrichment of the top 20 DEGs

Analysis of RNA-seq data using the edgeR package identified 1741 upregulated (including Lcn2, Cdkn1a) and 1815 downregulated (including Cyp1a2, Gsta4, Cyp2c54) genes in the CLP group compared with the Sham group when applying selection criteria of log2 FC ≥ 1 with statistical significance (*p* < 0.05) as shown in Fig. 2A and Fig. 2B. Comparative expression profiling between experimental groups was graphically represented through the heatmap in Fig. 2C and Supplementary Figure S1 (top 100 genes). GO enrichment analysis of DEGs revealed predominant association with biological functions including oxidative pressure, lipid metabolic process, protein binding, heme binding, iron ion binding, inflammatory response, xenobiotic metabolic process and innate immune response (Fig. 2D). Complementary KEGG pathway analysis demonstrated significant enrichment of DEGs in primary pathways such as steroid hormone biosynthesis, metabolism of drug by cytochrome P450, retinol metabolism, bile secretion, oxidative pressure, complement and coagulation cascades, ascorbate and aldarate metabolism, TNF signaling pathway, PPAR signaling pathway and srachidonic acid metabolism (Fig. 2E). Overall, these are closely related to oxidative pressure, liver metabolism, immune responses and inflammatory responses.

### Functional enrichment analyses of the role of genes modified by the difference m6A peaks

**Fig. 3:**
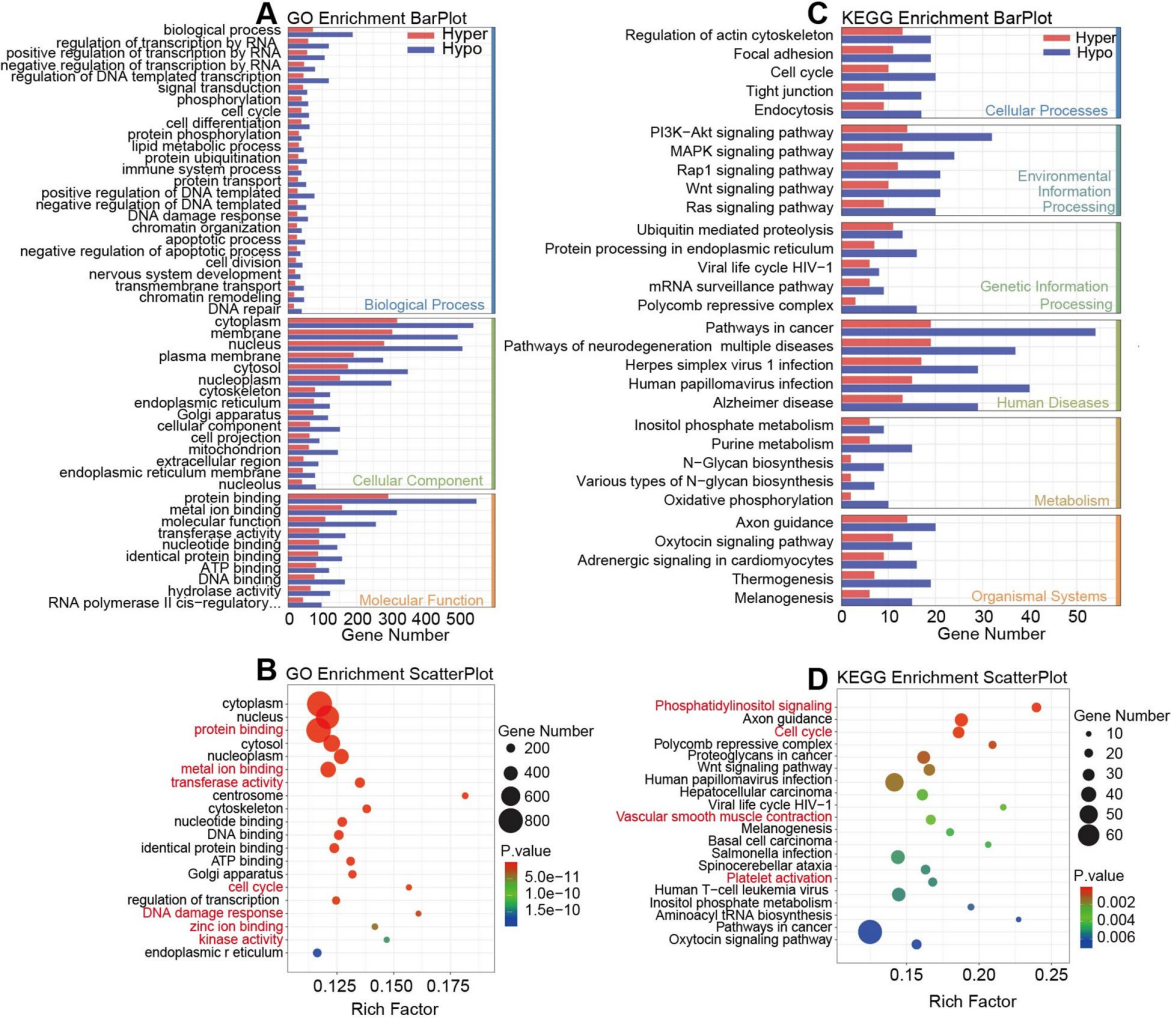
Functional enrichment analyses associated with m6A methylation modifications. **A** Bar graph of upregulation and downregulation of genes modified by difference m6A peak methylation modification through GO enrichment; **B** GO enrichment bubble map of genes modified by difference m6A peak methylation modification; **C** Bar graph of upregulation and downregulation of genes modified by difference m6A peak methylation modification through KEGG enrichment; **D** KEGG enrichment bubble map of genes modified by difference m6A peak methylation modification

To explore the pathophysiological significance of m6A modifications, we conducted both GO and KEGG analyses. According to the GO analysis, the upper m6A peak regulation was significantly associated with protein binding, biological processes, cytoplasm, etc. In addition, the lower m6A peak regulation exhibited significant correlations with protein binding, biological processes, regulation of DNA transcription in the cytoplasm, and so on (Fig. 3A). The GO Enrichment ScatterPlot showed that the most difference m6A peak-modified genes in the liver tissue of mice with sepsis were significantly enriched in protein binding, metal ion binding (zinc ion), enzyme activity (transferase, kinase), cell cycle and DNA damage response (Fig. 3B). The KEGG analysis revealed that the upregulation of m6A peaks was significantly associated with PI3K-Akt signaling pathway, MAPK signaling pathway, cell cycle, etc. Conversely, the downregulated m6A peaks were significantly associated with PI3K-Akt signaling pathway, cell cycle, and so on (Fig. 3C). The KEGG Enrichment ScatterPlot revealed that m6A modifications played a role in regulating various biological processes with widespread effects, primarily including phosphatidylinositol signaling system, cell cycle, vascular smooth muscle contraction and platelet activation (Fig. 3D).

These results suggest that m6A modifications may affect cell survival, immune responses and inflammatory reaction by regulating the expression of specific genes.

### Combined analyses of MeRIP-Seq and RNA-Seq data

**Fig. 4:**
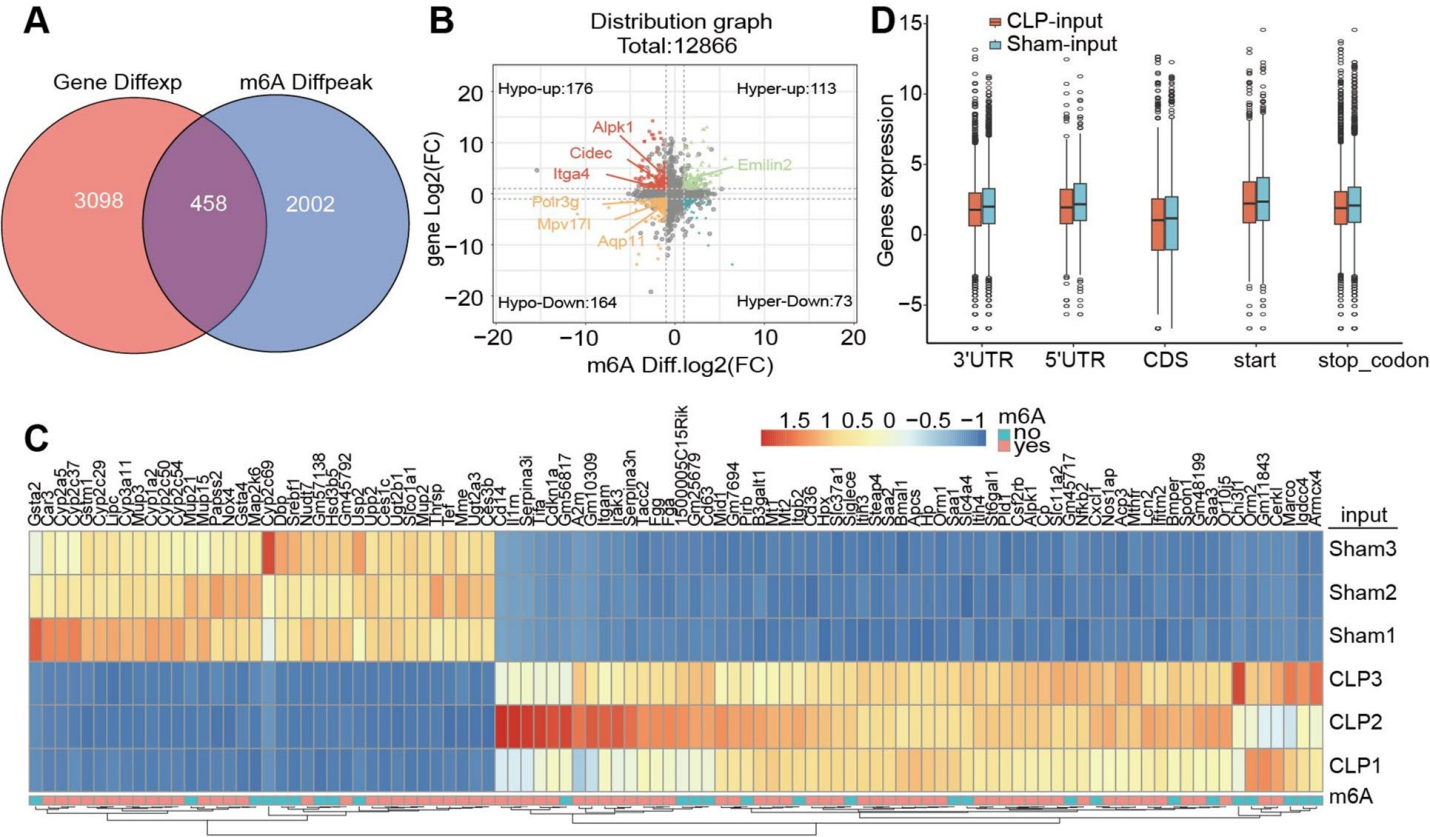
Combined analyses based on MeRIP-seq and RNA-seq data. **A** Venn diagram showed that 458 genes exhibited both m6A methylation and changes in transcription levels; **B** Four-quadrant diagram revealed the characteristic of m6A-modified genes changes; **C** Heatmap of m6A labeling of top 100 genes; **D** Box plot showed that the differences in the global expression level of m6A methylation-modified genes

To identify genes modified by m6A, we conducted combined analyses of MeRIP-seq and RNA-seq data and found that 458 genes exhibited both m6A modifications and changes in mRNA levels (Fig. 4A). Among these selected genes, 113 genes (Emilin 2) displayed increased expression with increased m6A methylation levels, whereas 176 genes (Alpk1, Cidec, Itga4) exhibited increased expression but decreased m6A methylation. Furthermore, 164 genes (Polr3g, Mpv17I, Aqp11) were downregulated with low m6A methylation levels, and 73 genes were downregulated despite increased m6A methylation levels (Fig. 4B). The heatmap illustrating top 100 genes is depicted in Fig. 4C. The overall expression level of m6A-methylated-modified genes on different functional elements of the gene was significantly different between the two groups (Fig. 4D).

### METTL3 has a significant impact on liver cell proliferation ability

**Fig. 5:**
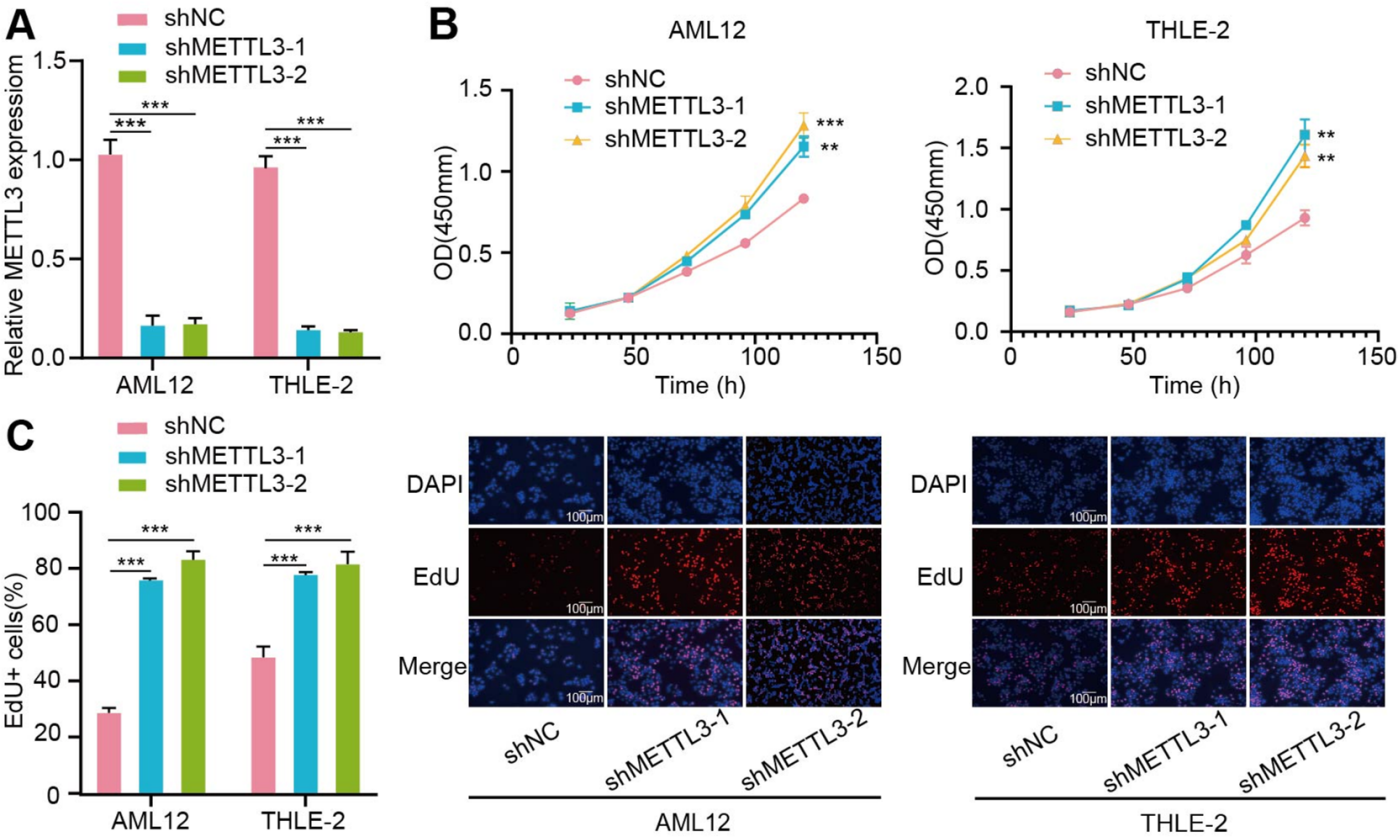
METTL3 has a significant impact on liver cell proliferation ability. **A** RT-qPCR was performed to access the knockout efficiency of METTL3 in AML-12 and THLE-2 cells; **B**, **C** CCK8 assay and EdU assay indicated that METTL3 enhanced proliferation ability of AML-12 and THLE-2 cells

In this study, we aimed to deepen our understanding of the role of m6A modifications in SILI and its underlying mechanisms by examining the effect of knocking down METTL3, a crucial regulatory factor of m6A, in AML-12 and THLE-2 liver cell lines. RT-qPCR results validated the efficient knockout of METTL3 in both cell lines (Fig. 5A). Subsequent CCK8 assay (Fig. 5B) and EdU assay (Fig. 5C) revealed a marked enhancement in the proliferative activity of AML-12 and THLE-2 cells following METTL3 knockdown. Collectively, these findings underscore the significant impact of METTL3 on the proliferation ability of liver cells, providing valuable insights into the regulatory roles of m6A modifications in cellular function.

## Discussion

As a complex inflammatory syndrome, sepsis is one of the leading causes of death in ICUs [1]. Although numerous studies have proposed diverse mechanisms for SILI, such as immune dysregulation, microcirculatory disruption, and the influence of inflammatory mediators, the molecular mechanisms underlying liver injury remain largely unknown [23]. In our research endeavor, we conducted an exhaustive evaluation of liver tissues obtained from mice subjected to CLP and Sham groups. This evaluation involved comparing the m6A transcriptome profiles of liver tissues between the CLP group and Sham group. We also examined the important disparities in various m6A modified by genes between the two groups and explored the regulation of m6A modifications at the RNA level, along with its association with liver injury. Mice treated with CLP exhibited prototypical sepsis symptoms, comprising lethargy, piloerection, and diarrhea. Serological analyses revealed a marked increase in serum AST and ALT levels in the CLP-treated mice, suggesting the occurrence and magnitude of liver damage. In addition to serological indicators, liver pathological examination has emerged as a pivotal tool for evaluating SILI. The liver tissue of CLP-treated mice exhibited characteristic pathological changes, including disorganization of tissue architecture, and interstitial edema. These pathological alterations were consistent with the elevated serum AST and ALT levels, thus further validating the presence of SILI. Our results are in good agreement with previous research, emphasizing the robustness of the model used in this study [24].

m6A modification represents one of the most widespread RNA modifications found in eukaryotes. It plays a vital role in regulating gene expression, mRNA stability, translation efficiency, and cellular functions. This particular modification occurs in a vast majority of eukaryotes [25, 26]. Our study showed that in the mouse liver tissue, the majority of m6A peaks are positioned within the 3′-UTR and CDS regions of mRNA. This observation emphasizes the essential function of m6A modifications in modulating mRNA stability, translation efficiency, and cellular performance [27].

In this study, the general extent of m6A alteration in the hepatic tissue of CLP-treated mice remained largely constant. However, significant variations were detected among genes with altered expression. For instance, GO enrichment analysis revealed that a considerable amount of these genes were concentrated in lipid metabolic process, protein binding, heme binding, etc. (Fig. 2D). The results of KEGG enrichment analysis suggested that genes with altered expression were primarily involved in steroid hormone biosynthesis, metabolism of drug by cytochrome P450, retinol metabolism, etc. (Fig. 2E). These results suggest that sepsis affects the metabolic and inflammatory responses of the liver by controlling the expression of particular genes [28, 29]. In addition, enrichment of DEGs in glutathione metabolism suggested their potential role in the antioxidant defense mechanism of the liver [30]. Moreover, GO enrichment analyses revealed that genes with differential m6A peak modification were primarily involved in protein binding, metal ion binding (zinc ion), enzyme activity (transferase, kinase), cell cycle, etc. (Fig. 3B) and KEGG enrichment analyses revealed they were associated with phosphatidylinositol signaling system, cell cycle, etc. (Fig. 3D). These pathways are vital in controlling cell survival, inflammatory responses, and immune responses [31]. This data emphasizes the vital role of m6A modification in SILI by modulating gene expression and mRNA stability, thereby influencing liver metabolic function and inflammatory responses [32, 33]. In addition, we also conducted motif analyses, which exposed the top three motifs of m6A alteration in two groups, providing a basis for understanding its impact on liver damage.

We employed GO and KEGG pathway enrichment analyses in this study to examine m6A-methylated gene functions. The results demonstrated a significant concentration of these genes within inflammation-related biological pathways, implying their potential involvement in sepsis-associated hepatic injury through regulation of inflammatory molecule expression. Notably, inflammatory cytokines including TNF-α, IL-1β, and IL-6 serve as critical regulators in sepsis pathogenesis [34]. These molecules drive the initiation and amplification of inflammatory processes by triggering sequential signaling events. The overproduction of inflammatory mediators can induce hepatocyte injury, manifesting as cellular necrosis, impaired mitochondrial function, and programmed cell death [35, 36].

Beyond inflammation and programmed cell death, the root causes of SILI also encompass oxidative stress and disruptions in mitochondrial function [37, 38]. In the context of oxidative stress, genes that have undergone m6A modifications are abundant in glutathione metabolism pathways, strengthening the antioxidant defenses of the liver through distinct mechanisms. For instance, m6A modifications possesses the capability to activate the Nrf2 signaling pathway, triggering the synthesis of numerous antioxidant enzymes. Furthermore, m6A modifications can alter oxidative stress levels of the liver by regulating the extracellular matrix and lipid metabolism, ultimately affecting the extent of liver cell damage [39, 40]. Regarding mitochondrial dysfunction, m6A-modified genes are prominently found in the cytoplasm, nucleus, and proteins, potentially affecting mitochondrial function and abundance by modulating the expression of genes involved in mitochondrial biosynthesis and energy metabolism. Modifications in mitochondrial function can directly influence cellular energy metabolism levels, thereby affecting cell survival and functionality. In addition, m6A modifications may regulate the redox reactions occurring within mitochondria, thereby affecting mitochondrial function and cellular metabolic state. Previous research has demonstrated that using bactericidal, nonbacteriolytic protein synthesis inhibitors reduces septic mortality [41]. The findings in this study provide novel perspectives on the molecular mechanisms underlying SILI and present potential pathways for the development of targeted therapeutic strategies [42].

Despite revealing the potential significance of m6A modifications in SILI, this study has several limitations. First, the limited sample size restricts the generalizability of the findings. Second, the study’s focus on a mouse model requires further verification for its applicability to human sepsis. Moreover, the exact molecular mechanism supporting m6A modifications remains incompletely understood, necessitating future in-depth investigations into the roles of m6A modifiers (including WTAP and IGF2BP2) in SILI. With the continuous development of technology, the drug discovery based on network pharmacology and omics technologies is undergoing a revolutionary change, and it is expected that targeted drugs for liver injury caused by sepsis can be developed in the future [43].

To summarize, the m6A transcriptome profiles between liver tissues of two groups and the unique m6A modification patterns in both groups revealed the crucial function of m6A modifications in liver injury of septic mice. The m6A modifications play a wide role in the pathophysiological mechanisms of the liver, involving in modulating cell survival, inflammatory reactions, and immune responses. These findings provide a novel insight into the mechanism underlying SILI and may pave the way for developing innovative therapeutic approaches.

## Supplementary Information

Supplementary material 1.

## Data Availability

No datasets were generated or analysed during the current study.
