# Supplementary figures and images for: Construction of RNA m6A profiles in liver tissue of mice in sepsis-induced liver injury based on m6A MeRIP-seq and RNA-seq
Source: Eur J Med Res. 2025 Aug 8;30:723. doi: 10.1186/s40001-025-02985-7 (PMC12333209; doi:10.1186/s40001-025-02985-7)

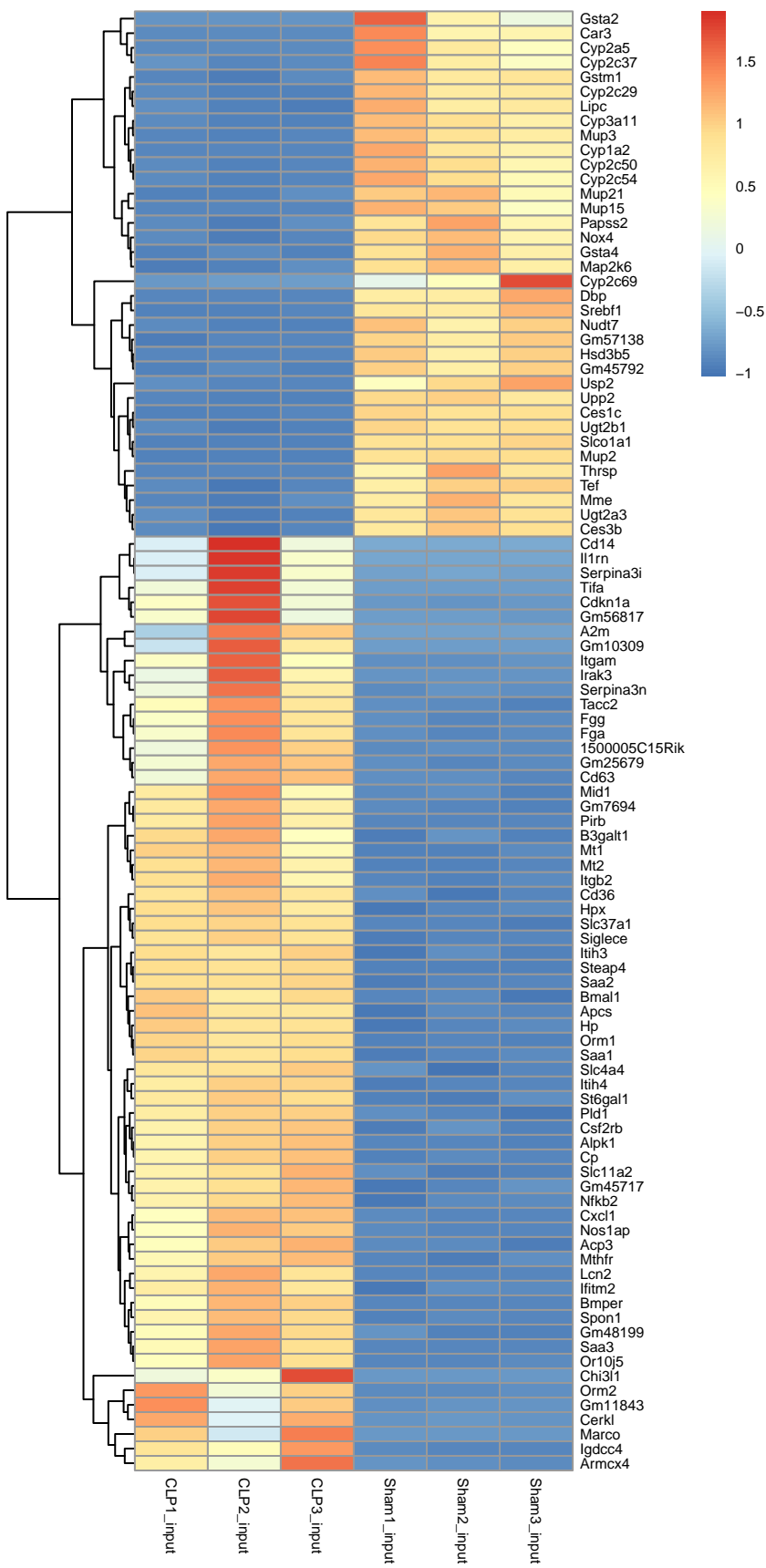

Supplement: Supplementary file 1 — Supplementary material 1. [file 40001_2025_2985_MOESM1_ESM.pdf]
